# Aging in place or aging out of place? Family caregivers’ perspectives on care for older Pakistani migrants in Norway

**DOI:** 10.1007/s10433-024-00832-y

**Published:** 2024-11-11

**Authors:** Sunita Shrestha, Sanjana Arora, Alistair Hunter, Jonas Debesay

**Affiliations:** 1 Department of Nursing and Health Promotion, Faculty of Health Sciences, Oslo Metropolitan University, Oslo, Norway; 2 Centre for Intercultural Communication, VID Specialized University, Stavanger, Norway; 3 School of Social and Environmental Sustainability, University of Glasgow, Dumfries, UK

**Keywords:** Family caregiver, Migrant, Aging, Informal care, Formal care, Trust

## Abstract

The aging population in Europe is increasing, with growing ethnic diversity among older individuals due to migration. Public policies emphasize ‘aging in place’ to address financial challenges and reduce the burden on the healthcare system. However, research often overlooks the heterogeneity among older people, especially non-European migrants. Also, research on family caregivers’ role in enabling ‘aging in place’ for older relatives with migrant backgrounds is limited, despite many older non-European migrants’ preference for family care in comparison to long-term institutional care. This study aims to explore the experiences and perspectives of family caregivers in the context of formal and informal care and their preferences regarding the place of care for older family members with migrant backgrounds, particularly the Pakistani Ahmadiyya community in Norway. Eighteen semi-structured individual interviews and two group interviews were conducted in Urdu and English with nineteen female family caregivers of older relatives, ages 25–62, residing in Norway. The analysis yielded three main themes: (1) “Nursing homes are not for the ones who love their parents”, (2) Preferences for home with the possibility of sharing care, and (3) Mismatch between family care needs and formal care. Family caregivers’ perception of health services has a decisive impact on their older relatives’ demand and use of formal health services, emphasizing the need for trust. Even though home care services were seen as a viable option, they should be adapted so that the family caregivers can use them and feel supported in enabling ‘aging in place’ for older migrants.

## Background

The aging population in Europe is on the rise, and increased migration has contributed to greater ethnic diversity in Europe’s older populations (WHO 2018). Older migrants, especially those aged 65 and above, are becoming more prominent in various countries, presenting specific healthcare needs (Northwood et al. 2018). In Norway, the proportion of migrants in the population aged 70 years and older is expected to rise from 5% in 2020 to approximately 25% in 2060 (Dzamarija 2022). Norway’s welfare state, funded through income taxation, ensures universal access to health and care services, allowing older adults to receive assistance when needed (Bjerke 2020; Helsedirektoratet 2018).

To address the financial challenges posed by an aging population and reduce the burden on healthcare systems, public policies on eldercare emphasize the concept of ‘aging in place’ (Helsedirektoratet 2017; Pedersen et al. 2024). This policy encourages older adults to live independently in their own homes, or with family (Cutchin 2003), receiving necessary care in a familiar environment rather than an institutionalized setting (Sampaio and Walsh 2023). ‘Aging in place’ emphasizes older adults’ needs and preferences, contributing to well-being, autonomy, and a sense of belonging in their communities (Lewis and Buffel 2020; Redzovic et al. 2023; Sampaio and Walsh 2023). Thus, age-friendly environments, encompassing physical and social aspects, are increasingly recognized as essential to meet the needs of the aging population (Lewis and Buffel 2020). ‘Aging in place’ policies view care as a shared responsibility, relying on active roles from family members to support older adults living at home and within their communities for as long as possible (Bjerke 2020; Helsedirektoratet 2017; Vangen and Herlofson 2023).

Family caregivers play a central role in enabling ‘aging in place’ as they assist with daily activities, emotional support, and navigating complex healthcare and social service systems (Schulz et al. 2020). Caregivers often experience ambivalent feelings towards care receivers (Pillemer et al. 2019). Despite satisfaction and closeness with care receivers, caregiving can come at a personal cost, impacting the caregivers’ health and social circles (Schulz et al. 2020; Stenberg and Hjelm 2022), especially among women and migrant caregivers due to the gendered nature of family care (Shrestha et al. 2023; Zygouri et al. 2021).

Studies indicate that relatively few older people with migrant backgrounds in Europe reside in long-term care facilities, and they tend to have higher expectations regarding family care^1^ from children (Horn 2023; Sagbakken et al. 2018; WHO 2018). Such expectations often arise due to cultural norms from the place of origin (Sun 2023), lack of access to formal care, limited language skills and poor health literacy among older migrants and their family caregivers (Debesay et al. 2022; Shrestha et al. 2023) as well as concerns about the potential for discrimination or prejudice in institutional care (Scott et al. 2022). Moreover, the lack of trust in formal care creates a distance between health personnel and family caregivers, hindering communication and collaboration, and often leading to lower utilization of healthcare services as family caregivers may hesitate to express their needs (Ahmad et al. 2023; Conradsen et al. 2024).

As an alternative to institutional care, migrants may opt for home care services, although they still represent a small minority in this sector (Næss and Vabø 2014; Scott et al. 2022). In home care services, family caregivers’ capital in regard to linguistic abilities and cultural knowledge is considered crucial for providing culturally sensitive healthcare (Gulestø et al. 2021). Despite the significant role of family caregivers in eldercare (Helsedirektoratet 2017, 2018), collaboration between them and healthcare personnel is often lacking (Häikiö et al. 2020). While changes in population composition demand increased competencies among health personnel, limited competency in addressing diversity is frequently reported (Ahmad et al. 2023; Debesay 2022; Gulestø et al. 2020).

Research on family caregivers’ roles in enabling ‘aging in place’ is limited but crucial. The heterogeneity among older people is rarely prioritized in research on ‘aging in place’ and migrant groups are often overlooked (Sampaio and Walsh 2023). Social policies for older people are often premised on gerontological theories of continuity and aging-in-place, but these frameworks may need to be revised given the significant and increasing proportions of the older population who have experienced geographical discontinuity and multiple places during their lifetimes (Torres and Hunter 2023). There is a need for more knowledge regarding older migrants’ preconditions for adequate care in their preferred place. Understanding family caregiver preferences can provide support that aligns with cultural, linguistic, and familial needs, thereby avoiding greater burden on institutional care in the long term. Hence, the closer involvement of family caregivers in a migration context also needs further exploration (Ahmad et al. 2023; Bjerke 2020). Therefore, this study aims to explore the experiences and perspectives of family caregivers in the context of both formal and informal care, and their preferences regarding the place of care for older family members with a Pakistani migrant background. The study focuses on the Pakistani Ahmadiyya community in Norway.

### Ahmadiyya community in Norway

In the late 1960s, many Pakistanis came to Norway as labor migrants, followed by their family members (Brochmann and Kjeldstadli 2008). Pakistanis constitute one of the largest non-European migrant populations in Norway, estimated at 40,000 (Ingebretsen et al. 2015). Among them, the Ahmadis, a religious minority primarily of Pakistani origin, number approximately 1200–1700 (Bisgaard 2010; Høydal et al. 2023). The Ahmadis arrived in the 1970s as refugees due to religious persecution by mainstream Sunni Muslims in Pakistan, making them a ‘minority within a minority’ in Norway (Jacobsen et al. 2015). Mainstream Muslims often exclude Ahmadi voices when representing Islam (Naserah 2022), and discriminate against Ahmadis even in Norway (Færseth 2021; Naserah 2022). Ahmadis’ narratives of persecution have contributed to their image as ‘good Muslims’ in the Western world (Yaqin et al. 2018). While Ahmadis prioritize integration, they maintain their culture, traditions, and religion through a close-knit community based on religious beliefs (Ahmed-Ghosh 2004; Appleyard 2015).

### Long-term eldercare services in Norway

Municipalities in Norway are responsible for long-term care services for older adults, which include home-based care and institutional care, with the latter reserved for those whose needs cannot be met through home-based care (Hellzén et al. 2024). Municipal service allocators assess the need for long-term care services, determine the appropriate services, and prioritize recipients when resources are limited (Christensen and Wærness 2017; Pedersen et al. 2024). Nursing homes provide housing options for individuals with special support and service needs, either municipally owned or as tenant-owner associations.

Home-based services are divided into two categories: home health care and home help. Home health care, typically provided by registered nurses, includes medication management, wound care, and observations, with 24-h care provided based on individual needs (Hellzén et al. 2024). Home help focuses on practical assistance, such as cleaning and grocery shopping, provided by nursing assistants. Additional services include provision of meals, alarm systems, respite care, home counseling, occupational therapy and physiotherapy (Hellzén et al. 2024; Næss and Vabø 2014). Although the Norwegian government has emphasized the importance of supporting family caregivers of older adults, comprehensive support in the form of care allowances and paid leave remains limited (Gautun and Bratt 2024).

## Trust and distrust in institutions

In our study, we use Luhmann’s theory of trust (Luhmann 1979, 2017) to make sense of the perceptions and care preferences of family caregivers, particularly concerning the choice of place of care for their older family members.

Luhmann’s trust theory distinguishes between two levels of trust: personal trust and system trust. Personal trust is crucial for individuals as it helps simplify their understanding of the world, enabling them to make informed decisions and take action. System trust, on the other hand, is essential for the proper functioning of social systems, as they need to manage complexity and build trust to operate effectively (Luhmann 1979, 2017; Meyer et al. 2008). System trust and interpersonal trust stand in a complex relationship with one another, such that they can both substitute and complement each other (Kroeger 2012, 2019; Luhmann 2017). For example, trust plays a pivotal role in securing individual communication and dialogue, which is necessary for accessing and benefiting from healthcare services (Meyer et al. 2008).

According to Luhmann, familiarity is a prerequisite for trust. When individuals are in a familiar and safe environment with people they perceive as having good intentions, trust can flourish, reducing concerns and facilitating action. Trust serves as a fundamental mechanism for reducing complexity and uncertainty, particularly in unfamiliar or risky situations (Luhmann 1979, 2017). Conversely, in situations of uncertainty, the possibilities for action decrease, forcing individuals to allocate resources to manage risks (Gilson 2006; Luhmann 1979, 2017). Distrust, on the other hand, is a rational response when services fail to meet individual needs (Luhmann 1979, 2017). Social systems can develop trust cultures through forms of self-presentation and other symbolic expressions, which constitutively influence the trusting behavior of their members (Kroeger 2018; Luhmann 2017).

Trust in the healthcare system can be influenced by factors such as familiarity, language barriers, and perceptions of cultural competence among healthcare providers (Debesay 2022). When healthcare systems do not adequately address the needs of heterogeneous individuals or groups, it can lead to prejudices against the system, thus hindering health seeking behaviors of individuals as well as their collaboration/interactions with health personnel (Conradsen et al. 2024). Applying Luhmann’s theory of trust in our study, we aim to gain insights into how trust and distrust in the healthcare system shape perceptions and care preferences for place of care for their aging family members with migrant backgrounds.

## Methods

We employed an explorative study using semi-structured interviews (John and Creswell 2023) to explore family caregivers’ experiences and perspectives on how formal care and informal care may shape their preferences for place of care for their older family members.

### Participants and recruitment

Participants for the study were recruited in two phases from August 2021 to November 2022. Initially, 9 participants were purposively selected through key informants (committee members) at a women’s organization consisting of all female members above the age of 15 years*.* One of the participants was recruited directly by the first author, contacting her through social media (Facebook). Later, the remaining nine participants were recruited using a combination of key informants (4 participants) and snowball sampling (five participants). The participants in our study were only women, between the age of 25–62 years old, who provided unpaid care to an older family member and had at least six months of experience as caregivers.

To capture the diversity of care experiences and perspectives, participants with varied migration histories, ages, educational backgrounds, marital statuses, family sizes, generations as well as parents (care receivers) with different migration histories were prioritized. The participants’ characteristics are shown in Table 1.

**Table 1:** Characteristics of participants

1. Age (in years)	No. of participants	4. Employment status	No. of participants
20–30	2	Homemaker	4
30–40	2	Part-time	4
40–50	7	Full-time	10
50–60	5	Unemployed	1
60–70	3		
2. Marital status		5. Care receivers	
Married	11	Mother	5
Unmarried	2	Father	1
Divorced	5	Mother-in-law	2
Widowed	1	Both parents	6
		Both parents-in-law	3
		Elder sister	1
		Grandparents	1
3. When migrated to Norway		6. Care model	
Migrated in adulthood	9	Co-residence	5
Migrated in childhood	5	Rotational care (older adults take turns at children’s houses)	5
Born in Norway	5	Rotational care (children stay nearby)	9

### The interviews and observations

The first author conducted data collection through 18 semi-structured individual interviews; 16 individual and 2 follow-up interviews. Additionally, two group interviews were conducted, each involving two participants. Notably, in group interviews, participants were siblings or relatives (sister and sister-in-law), and they were less vocal in group interviews regarding familial conflicts related to unequal contributions to care. However, these group sessions offered valuable insights into the intricate negotiations among family members concerning the allocation of responsibility for providing care to older family members.

In total, 19 female family caregivers of older Pakistani relatives, including daughters, daughters-in-law, a sister, and a granddaughter, were interviewed. The interviews employed a semi-structured guide designed to explore the care preferences among family caregivers of older migrant parents. Key questions in the interview guide included inquiries about the caregivers’ experiences with care services, suggestions for improving current care responsibilities, needed support for continuing family caregiving, preferences in caring for their parents, and satisfaction with the support received from Norwegian care services.

Both face-to-face interviews and online interviews were conducted based on participants’ preferences. Approximately half of the interviews (12) took place at a mosque, while the remainder occurred in public libraries (4) and via video calls on Teams (4). In addition to the formal interviews, the primary author actively participated in events organized at the mosque, facilitating informal conversations with women and providing valuable insights into the Ahmadiyya community. All the interviews were conducted by the first author in Urdu or English and were audio-recorded and later transcribed. Urdu interviews were translated verbatim into English. The duration of each interview ranged from 45 min to 3 h, accumulating to a total of 38 h and resulting in 375 pages of transcribed material.

### Data analysis

A reflexive thematic analysis, following Braun and Clarke (2022) was employed to analyze and generate themes in the dataset, as outlined in Table 2. The analysis encompassed six phases, with the first phase involving the familiarization of each transcript. The primary author immersed herself in the dataset by repeated readings of the transcripts and attentive listening to the audio recordings, noting immediate thoughts and ideas. Simultaneously, two reference transcripts were shared among co-authors to facilitate collective familiarization with the data. The second phase involved code generation. Initially, all authors convened to collaboratively generate codes from the reference transcripts. The first author then coded the remaining transcripts and organized the data around similar meanings. This organized data was subsequently shared with and discussed among the co-authors. In the third phase, codes were systematically organized according to potential themes related to the study’s aim, which focused on family caregivers’ preferences of care services. These themes underwent further discussion among the authors. Moving to the fourth phase, these themes were further reviewed and refined.

**Table 2:** The phases of thematic analysis (Insert here)

1. Familiarizing with the data	2. Generating initial codes	3. Searching for themes	4. Reviewing themes	5. Defining and naming themes	6. Producing the report
“Nurses could come to clean her up around 9–11 am, but her routine is she wakes up at 06:00 am, so from 6–7 am, I clean her up. When the nurses come then at that time, she is usually sleeping. Then I said them no, not to disturb her sleep.”	Option to say no Flexible routine	Flexible routine at home	Perceived preferences of seniors for staying at home	Preferences for home with the possibility of sharing care	Explaining ‘Preferences for home with the possibility of sharing care’ in findings section
"If we expose ourselves physically by being there for them for everything, then we will not be there for them psychologically. Having some help from a nurse, that is good. Otherwise, it is not easy to take 100% care responsibility for caring for kids, husband, and being an employee."	Preferences for sharing care with health professionals Difficult to take care responsibilities alone	Want to share care and still be a part of it	Preference of sharing care with health professionals		

In the fifth phase, the themes were redefined to ensure comprehensive coverage of the data and its alignment with the research question. The final phase involved producing the report (Braun et al. 2022). The data analysis process was characterized by an iterative and reflexive approach, combining both inductive and deductive methods. Table 2 delineates the phases of the thematic analysis, offering a transparent representation of how one of the themes in the study was derived. Through collaborative discussions, all authors contributed to constructing, defining, and refining the themes until a consensus was reached, enhancing the study’s rigor and trustworthiness.

### Ethical consideration

The study was reported to the Norwegian Center for Research Data (NSD). Prior to their involvement, all participants were duly informed about the study and explicitly notified that they retained the right to withdraw at any point, without the obligation to provide a reason. Written consent was obtained for face-to-face interviews, while verbal consent was secured for interviews conducted on Teams. Throughout the research process, the anonymity of participants was maintained in all transcripts and in the presentation of the results.

## Results

The analysis yielded three main themes: (1) “Nursing homes are not for the ones who love their parents”, (2) Preferences for home with the possibility of sharing care, and (3) Mismatch between family care needs and formal care.

### Nursing homes are not for the ones who love their parents

The study participants exhibited strong care ideals during our interviews, with the reference point for care norms being Pakistan, where it is considered a cultural and religious obligation for families to assume the responsibility of caring for their older adults at home. Consequently, reluctance towards institutionalized care was common among caregivers, insofar as nursing homes were perceived as inappropriate for their older family members. Loneliness in such facilities, particularly due to missing family members, was a recurring concern expressed by participants. This fear of separation led some older adults with severe health needs to conceal their health issues, fearing admission to nursing homes and being separated from their families. Further, some participants emphasized the need for residents to have private spaces, where regular family visits or occasional stayovers could be accommodated to address the perceived lack of companionship*.*

“*Old age homes should be more like apartments, not just a bedroom. That would be ideal because then I, my children, and my brother could visit him all the time and stay over for the night if he is unhappy or feeling lonely*.” (Participant 5, age 50–60, migrated to Norway during childhood)

Several participants highlighted the importance of dignified care, encompassing aspects such as food, daily activities, Pakistani TV programs, prayer, and gender-matching services for older adults, which were perceived to be lacking in nursing homes. Significant challenges for older adults of Pakistani background include language barriers and unfamiliarity with the Norwegian lifestyle encompassing daily routines, mealtimes, and social activities, which often do not align with their cultural and religious preferences and practices. These challenges were even more profound among so-called ‘zero generation’ migrants (Nedelcu 2023) who migrated later in life to reunite with their adult children in Norway.

Choosing nursing homes was commonly associated with relinquishing family responsibilities and seen as abandonment by caregivers. The negative perceptions surrounding nursing homes, including disrespectful treatment, inadequate care, and older individuals dying alone, were widely circulated within the Ahmadiyya community. Even caregivers employed in nursing homes expressed their reservations and had a generally poor impression of such facilities and health personnel working there.

“*They* [health personnel working in nursing homes] *don’t have time to sit with old people and talk with them. I worked there for 3 months. I felt pain in my heart because they are so old, and nobody is talking with them which is not good for old people.*” (Participant 8, age 40–50, migrated to Norway in adulthood)

Nursing homes were often considered a last resort when alternatives were unavailable for older parents, for example when adult children were not residing in the same country. Participant 15, who was born and raised in Norway, mentioned, “*Nursing homes are not for the ones who love their parents.”* Meanwhile, preferences for nursing homes emerged when care was reduced to physical assistance, particularly when older adults were bedridden and unable to move. Interestingly, current caregivers expressed a preference for nursing homes for themselves later in life, citing their adaptation to the Norwegian lifestyle and reduced language barriers as factors that would make the experience less challenging than it was for their parents. A participant explained this as follows:

“*It might be different for the next generation [our children’s generation] because their parents are active with their jobs, know the system, can speak* [Norwegian] *language, can drive and have the solid background* [economically sufficient]. *They will never ask us if we have to go to the doctor. They will not do such things for us as we do for our parents. They know we can do it ourselves.*” (Participant 9, age 40–50, born in Norway)

The participants’ perceived lack of barriers in navigating formal care for themselves and being more independent made them feel that their children would not feel the same obligation to provide care as they did for their parents.

### Preferences for home with the possibility of sharing care

Family caregivers played a multifaceted role in providing comprehensive support to older adults, encompassing physical, medical, emotional, and economic dimensions. Everyday responsibilities included assisting with tasks such as cooking, cleaning, shopping, and personal hygiene. Caregivers also took charge of coordinating medical appointments, facilitating transportation, and acting as intermediaries between the healthcare system and their older family members. Caregivers performed a key role in all such tasks, irrespective of the duration of residence of older migrants and their familiarity with the Norwegian healthcare system. Beyond practical assistance, caregivers served as companions, offering emotional support. Although older adults maintained regular contact with their relatives and siblings in Pakistan through phone and messenger calls, their social lives in Norway were limited, especially that of female older adults, which mostly constituted a few of their next-of-kin in Norway. Thus, family caregivers felt the pressure to entertain them as older adults rarely have activities to do outside of family.

While multi-generational living arrangements were generally viewed as fostering close-knit relationships and carrying higher prestige in their ethnic community, both in Norway and Pakistan, caregivers expressed concerns about the lack of physical and private space. Practical challenges due to lack of sufficient space were perceived to affect the overall caregiving dynamic. Moreover, caregivers’ preference for keeping older parents at home was juxtaposed with the challenges arising from the absence of age-friendly multi-generational housing structures. Affordability and the availability of suitable apartments with separate rooms and bathrooms for parents were perceived as challenging.

“*I do not bathe her at my place. We do not have a bathroom downstairs with room for my mother. When she goes to my sister’s place after 4–5 days, she* [her sister] *gives her bath there.*” (Participant 1, age 50–60, migrated to Norway in adulthood)

Notably, not having separate bathrooms for the older family members was a major issue for some, compromising hygiene and privacy.

“*In the case of my mother, we have to take care that the bathroom is empty when she needs to use it. Because she doesn’t have much control over her organs and her clothes will be ruined as well.*” (Participant 7, age 60–70, migrated to Norway in adulthood)

These examples shed light on the practical difficulties caregivers encounter, emphasizing the need for accessible and accommodating living spaces to facilitate caregiving. Despite their commitment to caregiving, family caregivers expressed uncertainties about their capacity to continue caregiving responsibilities in the future. These concerns were especially pronounced among those who were in full-time employment, leading to responses like *“I do not know”* when contemplating the future of family care. Aging caregivers facing health issues of their own found fulfilling care responsibilities increasingly challenging. This prompted a shift towards greater flexibility in adhering to family care norms, with caregivers tentatively exploring the possibilities of continuing informal caregiving.

Many caregivers echoed the preferences of older adults for remaining at home, aligning with their parents’ expectations. They emphasized the comfort of a familiar environment and frequent visits from relatives. Also, having parents at home allowed caregivers to check on them regularly and maintain a flexible routine that accommodated the older adults’ preferences for daily activities, food, and sleep in a more relaxed way, unlike what was perceived to be the norm in nursing homes. Additionally, some caregivers advocated for sharing care responsibilities with home care services, allowing them to have more control over the care schedule. For example, participant 1 favored having an option to say ‘No’ to home care services when they did not align with the schedule of the older adults.

“*Nurses could come to clean her up around 9–11 am, but her routine is she wakes up at 06:00 am, so from 6–7 am, I already clean her up. When the nurses come then at that time, she is usually sleeping. Then I said to them ‘No’, not to disturb her sleep.*” (Participant 1, age 50–60, migrated to Norway in adulthood)

A few participants who were using home care services expressed the importance of being physically present, especially for older family members with limited language skills, in order to facilitate communication and to provide comfort during interactions with healthcare professionals. Participant 6, age 50–60, migrated to Norway in adulthood, explained, for example, that her mother-in-law often asked her to stay in the room while nurses cleaned her and changed her clothes. She added, *“She feels comfortable because she does not know nurses but knows me.”*

Many caregivers expressed a preference for home care services, viewing them as a more favorable alternative where the care burden is shared with health professionals. Also, it reflected a pragmatic approach to balancing their multiple roles:

“*If we expose ourselves physically by being there for them for everything, then we will not be there for them psychologically. Having some help from a nurse is good. Otherwise, it is not easy to take 100% responsibility while also caring for kids and being an employee.*” (Participant 13, age 40–50, born in Norway)

While home care nurses were expected to manage personal hygiene, family members acknowledged their continued responsibility for other aspects of caregiving. This nuanced division of responsibilities highlighted the complexity of caregiving within a dual context: framing eldercare as a family responsibility on one hand, and as a state responsibility on the other. Collaboration with professionals was sought, yet certain tasks remained under the familial domain. Some participants revealed a hesitancy to openly discuss the use of home care services, fearing potential judgments from others about their commitment to parental care. For instance, participant 5 exemplified this concern, noting the societal expectation, particularly for daughters, to provide essential care like food.

“*If you are a daughter and do not provide food, then people would say something is wrong with you. I mean, can’t you even make food for your own dad? The other thing is OK because we understand that personal hygiene is something that your parents will not be comfortable with you.*” (Participant 5, age 50–60, migrated to Norway in childhood)

Even when utilizing home care services, caregivers emphasized the importance of demonstrating their active involvement in caregiving to meet societal expectations. Conversations within the community, often occurring informally at places like the Mosque, predominantly revolved around medical assistance (regular check-ups with general practitioners), social benefits (transportation to and from the Mosque), and equipment (wheelchair and stair lifts) that could ease their daily activities outside institutional care. However, participants articulated that there was a notable absence of discussions about alternative forms of care, such as nursing homes or home care services. This suggests a potential stigma or lack of openness regarding these alternative care options within the community.

### Mismatch between family care needs and formal care

When queried about the support they need from formal care, many family caregivers were unsure, alluding to the normalization of caregiving where the thought of seeking help rarely crosses their minds, and perceived it as a natural part of their daily life.

“*We have never thought about the question you have just asked. Like if I am taking care of my mother and this is also 24 h work, but we have never thought about it in our mind what should be there for us.*” (Participant 7, age 60–70, migrated to Norway in adulthood)

A prevalent issue emerged concerning caregivers’ limited awareness of their rights and available services. Even highly educated participants, including those born and raised in Norway, struggled to navigate the formal care system. Participant 5 (age 50–60, migrated to Norway in childhood), illustrated this by acknowledging the potential benefit of respite care so that she can go on holidays with her children but remained uncertain about how to access it. Similarly, many other participants, even those working as health professionals, emphasized the need for clarity on accessing economic support as informal caregivers.

“*My father says that there must be a system where help is provided to children who want to take care of their parents. I think this is a better option. If a person is looking after their parents, then support them instead of making it difficult for them to keep their parents. If I do 50% of the work and if I get 50% help, not even 50%, but a little help to make things work out, then I can take care of my mum.*” (Participant 15, age 20–30, born in Norway)

Overall, all the participants in the study, despite their limited knowledge of available care services, consistently perceived higher quality of services in Norway as compared to Pakistan. They particularly appreciated the free health care services and the provision of free medical equipment for older individuals. Though many family caregivers expressed their reluctance to be identified as Ahmadis among other Pakistani, they trusted that services are delivered equally to everyone, regardless of their ethnic and economic background.

“*You know in our countries *[referring both Pakistan and Nepal, as first author’s country of origin is Nepal]*, there is not much help if you are not rich but in Norway, the difference is that you can get your rights. I have never heard anyone saying that their parents were not given the rights they deserved from the municipality or government or hospital because we are Pakistani. (…) It is not in the system here that it is necessary to reveal your religious identity to doctors, but if someone already knows about you then they *[health personnel]* do not keep their grudges at profession, but if it was in Pakistan, it would have been different.*” (Participant 9, age 40–50, born in Norway).

Though the participants expressed confidence in receiving equal services for their older family members, firstly, still, many shared their experiences of their older parents not getting appropriate help when they did not assist them to doctor appointments, particularly due to language barriers. Secondly, they were skeptical about the support they could receive as informal caregivers. They also spoke about the health system’s attention being solely on patients (older adults) and not acknowledging the efforts or needs of those who take care of them at home. In a group interview, participants expressed skepticism through satirical remarks, such as “maybe one-day spa! [both participants laugh]” an ironic statement, suggesting the family caregivers’ lack of trust in receiving substantial support. Such attitudes were shared by other participants in the individual interviews where they firmly believed that the health care system neither recognizes their contribution to eldercare nor addresses their health needs as caregivers. For example, participant 7 articulated a common belief that seeking help, especially as a caregiver, might be met with resistance from the state.

“*If I go to the municipality right now and tell them I need something in return for taking 24-h care of my mother, then they will say, why did you keep her? You should send her to an old home. They will not give me any medal to praise my efforts in taking care. (…) If I have a psychological or health related problem then the system will help me* [as a citizen accessing individual health is my rights]*, but if I have a health issue due to caregiving, I don’t think they would help me much in this case.*” (Participant 7, age 60–70, migrated to Norway in adulthood)

The perception of a poor understanding of family caregiving by the health personnel discouraged some caregivers from seeking help. Participant 15 shared her experience of discontinuing psychological help due to a counseling professional’s lack of competency in addressing her caregiving-related issues adequately.

“*She advised me to tell my mum I needed time for myself* [laughs]*. I found it very strange because my mum is very sick, and how can I say such a thing to her? So, I stopped going there.*” -Participant 15

Based on her community care norms, where adult children are expected to look after their older parents, she felt that not taking care of her mother was hardly a viable option. However, this understanding was not reflected by the health personnel she was consulting. This showed a mismatch between the services offered and the nuanced needs of migrant-background caregivers, regardless of language proficiency. Similarly, preferences for same-sex caregivers in home care services further complicated the collaboration between informal and formal care. While older parents’ preferences were for same-sex nurses for personal hygiene assistance, such preferences were rarely accommodated, placing the caregiving burden back on family members. This exemplifies a situation where formal caregiving fails to align with the preferences and needs of the informal caregivers.

“*We had requested that no male nurse should be sent for her because my mother-in-law did not like that a male nurse would touch her. When they* [Home service nurse from municipality]* would call me to send male nurse, then I used to say that she will not even give him to enter her room, so instead I used to do her cleaning, changing diapers, and bathing.*” (Participant 6, age 50–60, migrated to Norway in adulthood)

The dilemma between formal and informal care was evident in cases such as Participant 1’s situation, where she shared caregiving responsibilities with her sister. In this shared care responsibility, their mother moved between their houses at certain intervals (every few days or every week), a strategy to distribute the care burden within the family system. However, this approach limits access to municipality services due to the geographical separation of the sisters, residing in different parts of Oslo Municipality. For instance, participant 1(age 50–60, migrated to Norway in adulthood) stated, “*My mother gets the physiotherapy while she stays at her* [her sister] *place, and they say that a single person cannot get those facilities at many places.”* This highlighted the potential for friction between formal caregiving structures and informal caregiving arrangements. Similar problems arose regarding economic help that family caregivers could get:

“*She* [home nurse] *was also mentioning about support money for family caregivers. If she* [mother] *only stays with her* [sister] *then she could fill in the form and submit it to municipality. Then she [her sister] gets money for taking care of our mother otherwise we both can’t claim for the money.*” (Participant 1, age 50–60, migrated to Norway during adulthood)

This mismatch between the family care needs and formal care resulted in the paradoxical situation whereby family caregivers have to choose either between the available formal support/services—on condition of becoming the primary caregiver—or sharing the care responsibilities with other family members at the cost of compromising formal care support.

## Discussion

The aim of this study was to explore the experiences and perspectives of family caregivers in the context of both formal and informal care, and their preferences regarding the place of care for older family members with a Pakistani migrant background in Norway. Our findings indicate that the family caregivers experienced an aversion towards nursing homes and were favorable towards home care services. Since none of the family caregivers in our study had yet placed their older parents in nursing homes, the study participants’ views seemed to reflect a general preunderstanding, while their perspectives on home-based services were more practice oriented as some participants had firsthand experience with these services. They also felt a lack of compatibility between their own care needs and what the public care services can provide.

Older migrants, similar to older adults in general, cite a strong preference for ‘aging in place’, primarily desiring to remain in their own homes. The inclination towards staying at home is consistent with findings from previous studies (Arora et al. 2020; Mellingen Bjerke 2017; Næss and Vabø 2014; Sagbakken et al. 2018). This is in line with our participants’ view that their older relatives’ preference for staying at home was so profound that some of the older migrants even preferred to hide their ailments, fearing that they might be made to live in nursing homes.

Migrant and diasporic communities often undergo distinct aging experiences, shaped by the concept of transnationalism, which involves connections to multiple places, communities, and homes (Levitt and Schiller 2004; Sampaio and Walsh 2023). While families living together in multi-generational arrangements are perceived as ideal within the Pakistani migrant community in Norway (Arora et al. 2020; Aarset 2015), this trend might be decreasing (Shrestha et al. 2024). It has also been found that they do not often desire to return to their country of origin in old age (Arora et al. 2020). This may be especially the case for older migrants from the Ahmadiyya community who have come to Norway as refugees and experienced being a religious minority in Pakistan (Appleyard 2015; Jacobsen et al. 2015). The perceived lack of good healthcare in Pakistan among the participants in our study also discouraged their return.

In our study, we observed that the sense of place among older migrants was primarily linked to the presence of family members rather than mere attachment to a specific physical location, a distinction often highlighted in the literature on ‘aging in place’ (Arias-Merino et al. 2022; Sampaio and Walsh 2023). This indicates a high level of interpersonal trust towards family caregivers as familiarity is a prerequisite for trust (Luhmann 1979, 2017) and highlights the significance of trust in shaping preferences for place of care. This is also exemplified through rotational care, where many older migrants prefer temporary living arrangements at their children’s homes, rather than opting for a permanent place of care away from their children. Trust can flourish and facilitate actions by reducing concerns when individuals are in a familiar and safe environment with people they perceive as having good intentions for them (Luhmann 1979, 2017). Thus, the presence of family members played a crucial role in shaping the sense of ‘aging in place’ for older migrants in this study.

Participants in this study appeared to hold a binary conceptualization of the primary place of care either living with children at home, or residence in a nursing home. Notably, family caregivers seemed to lack awareness of other options such as residential homes which can provide independent living or assisted living, as well as nursing care, which could place them at a disadvantage within the demand-oriented long-term care system (Ahmad et al. 2023). In institutionalized care settings such as nursing homes, everyday activities are often performed collectively with many other residents, and individuals may feel that they are treated equally and required to conform to a standard routine (Goffman 1961). In our study, the institutionalized care setting did not seem to align with the individual needs and preferences of older migrants’ caregivers, where a perceived unfamiliar environment compromised individuals’ trust in the health system (Luhmann 1979, 2017).

Thus, the perception of nursing homes was largely negative, with participants viewing them as places devoid of a homely atmosphere, culturally meaningful activities, and familiar food. They were also perceived as lacking a family presence and characterized by adherence to Norwegian norms and routines. In our study, compromised trust in nursing homes impacted how family caregivers perceived the competence of health personnel in addressing residents’ emotional needs, demonstrating the interplay between system trust and interpersonal trust (Kroeger 2012, 2019; Luhmann 2017). Care by family members in their own homes, thereby maintaining emotional support and connections to their culture and lifestyle, was integral in shaping their perceptions of nursing homes as places of poor care.

According to Luhmann’s trust theory (Luhmann 1979, 2017), distrust may arise as a rational response when services do not cater to individual needs. While the family caregivers’ accounts did not indicate rigid distrust towards nursing home services, they pointed towards their insecure expectations regarding formal care services in meeting the needs of their older family members. Concerns about nursing homes limiting older Ahmadis’ preferred lifestyles, care expectations, and connections with their families significantly contributed to prejudices and reluctance among participants. Research has shown that perceptions of care quality increase when person-centered care, i.e., care focused on residents’ preferences and well-being, is operationalized and takes precedence over routines and practical tasks (Grøndahl et al. 2018).

However, the complex system of residential care with its symbolic presentation as institutionalized places with rigid routines influenced the trust behavior of participants. These concerns align with research pointing out the underutilization of formal care services by older migrants and the necessity of addressing diversity (Ahmad et al. 2023; Horn 2023; Lillekroken et al. 2024). Caregivers’ perceptions of themselves as better suited for future residence in nursing homes, compared to their older family members, further highlights the lack of adaptiveness of health services to individual needs. When healthcare systems do not adequately address the needs of heterogeneous individuals or groups, it can lead to prejudices against the functioning of the care system, thus hindering equitable access to long-term care services (Conradsen et al. 2024; Luhmann 1979, 2017).

Older adults’ experiences of ‘aging in place’ vary considerably based on gender (perceived tradition of gender segregation *(purdah)* in Ahmadi Islam), class, race, and migration histories (Sampaio and Walsh 2023). Regardless, many non-European migrants may be more vulnerable as compared to the general population due to their lower socioeconomic status, language barriers, and limited access to appropriate health services (Debesay et al. 2023; WHO 2018). This vulnerability may increase their dependency on family members even more (Ajrouch et al. 2023; Sun 2023). Consequently, for migrant family caregivers in our study, ‘aging in place’ often entails intensive caregiving within a transnational care context, irrespective of the health needs of the older family members. This is because, even when home care services are utilized, younger caregivers frequently serve as intermediaries and are both expected and needed to be present during visits by home care professionals. Caregiving in this context encompasses not only physical tasks but also the provision of emotional and social support and companionship. The family caregivers’ responsibilities extend beyond routine tasks to include navigating the healthcare system on behalf of their older family members.

While some older migrants and their families may remain hesitant about using nursing homes and other specialized housing options, there is a growing indication that an increasing number of families with non-European background are becoming open to home care services in Europe (Horn 2023). Our study was consistent with this trend, as it reveals that family caregivers often desire home care services to help share their caregiving responsibilities with health professionals, particularly concerning physical assistance. This indicates that distrust towards formal care is not rigid or absolute, as they seem to have some interpersonal trust towards home care professionals. It is possible that this interpersonal trust towards home care professionals can be converted into trust in the formal care system as a whole (Luhmann 2017).

However, their reluctance to discuss the use of home care services within the community also points towards the role of group orientation in influencing trust (Luhmann 2017), where alternatives to family care are associated with stigma and shame within the community (Tavernier and Draulans 2018). Though home care services were trusted for physical assistance, they still lacked trust in providing emotional support. Families still perceived themselves as more appropriate to provide emotional care as well as assist in daily tasks, such as cooking their parents’ preferred food. Having a familiar face and help when communicating with homecare professionals, especially in cases where older adults had limited language skills, offered a sense of comfort to the older family members. This highlights that being a part of the care process, even when shared with formal care services, remains important and meaningful for family caregivers.

In contrast, a recent study in Norway among home care professionals (Gulestø et al. 2021) revealed that the presence of family members in care settings was often viewed as inappropriate and disruptive. This was especially the case when family members observed or monitored healthcare personnel’s work and expressed opinions about the type of care provided. Prior research has shown that health personnel often lack understanding about the care context of older adults with a migrant background (Ahmad et al. 2023). Involvement of family members can thus be vital to understanding and addressing older migrants’ care needs by home care professionals, especially as many caregivers mediate communication between the two. This involvement could also offer crucial insights into the care circumstances and challenges faced by family caregivers, potentially enhancing their well-being. However, home care professionals’ disapproval of family members’ involvement through symbolic gestures, may influence the trusting behavior of both family caregivers and older migrants, thus making them less likely to share their care needs and be able to access services in an equitable manner (Ahmad et al. 2023; Czapka and Sagbakken 2020).

Patient trust is often based on their perception of healthcare personnel’s competence and genuine interest in the patients’ well-being (Conradsen et al. 2024). The lack of mutual understanding due to limited competency among health personnel can create a fertile ground for mistrust between patients and healthcare personnel (Debesay 2022). This may result in family caregivers doubting the competency of health personnel in our study, hence influencing the help-seeking behavior of family caregivers and further distancing them from formal care. This disparity in perspectives on what constitutes appropriate healthcare between family caregivers and healthcare professionals can hinder interactions and collaboration between them, presenting a significant challenge for enabling ‘aging in place’ for older adults.

Our study also showed the perceived higher quality of eldercare services in Norway as compared to Pakistan. Migrants, especially the first-generation, tend to have higher levels of trust in public institutions than natives because they compare their experiences with public institutions in their home countries as their reference point (Röder and Mühlau 2012). However, such trust remains fragile and can be easily undermined by negative experiences, leading to distrust in the Norwegian welfare system (Bjerke 2020). While they seemed to indicate confidence in accessing services equally, they showed less confidence in their ability to receive services in an equitable manner. Trust and distrust towards formal care services was therefore complex and nuanced for family caregivers. Norway’s universalistic welfare system, while based on principles of equality, is also rooted in the dominant culture (Gulestø et al. 2021). Therefore, individuals from ethnic backgrounds with different care needs, sensitive to their culture, language, and circumstances, are sometimes viewed as too demanding (Ahmad et al. 2023; Gulestø et al. 2021).

Ahmad et al. (2023) demonstrated that narrow or prejudiced conceptions among healthcare personnel regarding the culture of individuals with a migrant background hinder a full understanding of their care context and needs, thereby reinforcing social inequalities and compromising person-centered care for older migrants. In our study, rotational care was a method of dividing caregiving responsibilities among family members. However, the geographical separation of family members and the complexity of accessing services during rotational caregiving underscored a disconnect between formal caregiving structures and the contextual realities of informal caregiving arrangements among migrant-origin families. Additionally, many older Pakistani migrants adhere to Islamic guidelines regarding interactions between the sexes (Shrestha et al. 2024), making it problematic for a care provider of the opposite sex to perform intimate care tasks (Bjerke 2020). This lack of gender-matching services often places the burden of care back on family members of older adults.

Using Luhmann’s theory of trust as an analytical lens, it becomes evident that the disconnection between formal and informal care further contributes to uncertainty among family caregivers. Trust in institutions serves to simplify complex situations (Luhmann 1979, 2017). The absence of trust created uncertainty, as participants lacked confidence in institutions’ ability to fulfill the needs of their older family members. Therefore, the continuation of family caregiving for ‘aging in place’ was characterized by uncertainty due to the lack of appropriate public health and social services for both older adults with migrant backgrounds and their family caregivers. This lack of support not only complicates caregiving but also exacerbates gender inequality within the Ahmadiyya community, where women predominantly bear the caregiving burden.

### Strengths and limitations

All the participants in the study had firsthand experiences of caring for their older parents, which is a notable strength as their perceptions and preferences were based on their personal experiences. We made deliberate efforts to include a diverse sample of participants based on factors such as education, age, employment, and linguistic proficiency, which yielded rich data. Conducting interviews in both Urdu and English allowed participants to comfortably share their experiences in their preferred languages. Group interviews with siblings or relatives (e.g., sister and sister-in-law) offered insights into intra-generational dynamics of shared care arrangements and facilitated discussions on the mismatch between family and formal care.

While face-to-face interviews are often regarded as ideal for establishing rapport and trust between interviewer and participant (John and Creswell 2023), online interviews also offered several advantages in this study. Online interviews helped us reach participants with digital competency and supported our deliberate strategy to conduct interviews outside the Mosque, when possible, thereby reducing potential location-related biases in the data. Additionally, offering the option of online interviews made it convenient for family caregivers who could not spare time for face-to-face interviews due to their caregiving responsibilities and full-time jobs. So, combining online interviews with in-person interviews helped to recruit even those participants who otherwise would not have been able to participate, given their time constraints, but wanted to share their experiences. Furthermore, the use of in-depth interviews and active listening during data collection enhanced the quality of the data, as participants had ample time to share their experiences.

However, it is important to acknowledge some limitations of our study. The focus was specifically on the Ahmadiyya community, which is a minority within a minority, and while this offers unique insights, it doesn’t represent the broader Pakistani population in Norway. We focused on adult children providing care to their parents, and the care arrangements for older adults without children were beyond the scope of this study. Also, incorporating the perspectives of older adults and healthcare professionals in home-care services could provide a more comprehensive understanding of the topic.

### Future implications

Understanding the meaning of care among migrant-background populations can inform health personnel, as well as policies and programs aimed at supporting them. Understanding the care context of individuals with migrant backgrounds could contribute to expanding the capacity of health personnel to address the diverse needs of ethnic communities, hence improving the collaboration between family caregivers and healthcare professionals. Furthermore, establishing trust-building measures in healthcare and social services can enhance the ability of migrants to ‘age in place’, which may be obtained through a person-centered approach for healthcare institutions (Ahmad et al. 2023; Olsen et al. 2019). By fostering better collaboration with family caregivers, healthcare institutions need to adopt culturally, linguistically, and context-sensitive person-centered practices, to assist family caregivers in caring for their older relatives, thereby encouraging safe aging-in-place opportunities for older migrants. Implementing targeted awareness programs to educate family caregivers about the available services for them and their older relatives could contribute to better navigation of demand-based formal care systems in Norway.

## Conclusion

The perceived lack of adaptation by the healthcare system, coupled with issues of accessibility and diversity considerations, highlights the need for a more nuanced and responsive approach in formal care to complement the diverse needs of older migrants and their family caregivers, crucial for enabling ‘aging in place.’ Therefore, this study has shed light on the roles of family caregivers in facilitating ‘aging in place’ among older adults with Pakistani migrant backgrounds in Norway by explaining the complex dynamics of formal and informal eldercare.

Family caregivers’ perception of health services may have a decisive impact on their older relatives’ demand and use of formal health services, which further highlights the importance of trust among family caregivers of older migrants. Questions of trust or distrust in relation to formal care services, along with concerns about diversity sensitivity and individual needs, profoundly influence the choices made by family caregivers. Even though home care services were seen as a viable option, it is important to adapt the services so that family caregivers can use them and feel supported as well as acknowledged in their crucial role in enabling older individuals with migrant backgrounds to ‘age in place’.

## Data Availability

The data supporting this study’s findings are available on request from the corresponding author. The data are not publicly available due to privacy or ethical concerns.

## Footnotes

1 In this paper, family care implies that care to older adults is given by a member of the family, either close or a distant relative, while informal care refers to the care given by individual of any age who provides unpaid assistance to a person in need of help.
